# Epistasis at the SARS-CoV-2 Receptor-Binding Domain Interface and the Propitiously Boring Implications for Vaccine Escape

**DOI:** 10.1128/mbio.00135-22

**Published:** 2022-03-15

**Authors:** Nash D. Rochman, Guilhem Faure, Yuri I. Wolf, Lydia Freddolino, Feng Zhang, Eugene V. Koonin

**Affiliations:** a National Center for Biotechnology Information, National Library of Medicine, Bethesda, Maryland, USA; b Broad Institute of MIT and Harvard, Cambridge, Massachusetts, USA; c Department of Biological Chemistry, University of Michigan Medical School, Ann Arbor, Michigan, USA; d Department of Computational Medicine and Bioinformatics, University of Michigan Medical School, Ann Arbor, Michigan, USA; e Howard Hughes Medical Institute, Massachusetts Institute of Technology, Cambridge, Massachusetts, USA; f McGovern Institute for Brain Research, Massachusetts Institute of Technology, Cambridge, Massachusetts, USA; g Department of Brain and Cognitive Sciences, Massachusetts Institute of Technology, Cambridge, Massachusetts, USA; h Department of Biological Engineering, Massachusetts Institute of Technology, Cambridge, Massachusetts, USA; Washington University School of Medicine

**Keywords:** SARS-CoV-2, Delta variant, Gamma variant, Omicron variant, escape mutants, epistasis, protein structure modeling, Rosetta

## Abstract

At the time of this writing, December 2021, potential emergence of vaccine escape variants of severe acute respiratory syndrome coronavirus 2 (SARS-CoV-2) is a grave global concern. The interface between the receptor-binding domain (RBD) of SARS-CoV-2 spike (S) protein and the host receptor (ACE2) overlaps the binding site of principal neutralizing antibodies (NAb), limiting the repertoire of viable mutations. Nonetheless, variants with multiple RBD mutations have risen to dominance. Nonadditive, epistatic relationships among RBD mutations are apparent, and assessing the impact of such epistasis on the mutational landscape, particularly the risk of vaccine escape, is crucial. We employed protein structure modeling using Rosetta to compare the effects of all single mutants at the RBD-NAb and RBD-ACE2 interfaces for the wild type and Delta, Gamma, and Omicron variants. Overall, epistasis at the RBD interface appears to be limited, and the effects of most multiple mutations are additive. Epistasis at the Delta variant interface weakly stabilizes NAb interaction relative to ACE2 interaction, whereas in Gamma, epistasis more substantially destabilizes NAb interaction. Despite bearing many more RBD mutations, the epistatic landscape of Omicron closely resembles that of Gamma. Thus, although Omicron poses new risks not observed with Delta, structural constraints on the RBD appear to hamper continued evolution toward more complete vaccine escape. The modest ensemble of mutations relative to the wild type that are currently known to reduce vaccine efficacy is likely to contain the majority of all possible escape mutations for future variants, predicting the continued efficacy of the existing vaccines.

## INTRODUCTION

When severe acute respiratory syndrome coronavirus 2 (SARS-CoV-2) first emerged as a global public health concern early in 2020, there was considerable debate regarding whether the low mutation rate of the virus and the relatively inflexible receptor-binding domain (RBD) of the antigenic spike (S) protein would admit robust host adaptation (1, 2). By 2021, it became clear that SARS-CoV-2 has access to a broad mutational repertoire enabling extensive diversification (3) and that without vaccination, SARS-CoV-2 would likely result in substantial global disease burden for a protracted period (4, 5). The development of multiple, effective vaccines against SARS-CoV-2 (6) makes it possible to dramatically reduce this burden. However, at the time of writing, December 2021, the majority of the global population remains unvaccinated as the Omicron variant is poised to replace the Delta variant as the dominant strain worldwide. Existing vaccine efficacy against the Omicron variant might be substantially reduced relative to the wild type (WT) (https://www.cdc.gov/coronavirus/2019-ncov/science/science-briefs/scientific-brief-omicron-variant.html), and the potential for continued evolution toward more complete vaccine escape (7) is a major global concern (https://www.cdc.gov/coronavirus/2019-ncov/variants/variant-info.html).

The interface between the receptor-binding domain (RBD) of the S protein and the host receptor (ACE2) largely overlaps the binding sites for the most potent neutralizing antibodies (NAb) (8, 9), limiting the scope of viable mutations. Nevertheless, multiple variants containing single mutations in the RBD that, to different extents, reduce NAb binding have begun to circulate (8–10). Moreover, variants with multiple mutations in the RBD have risen to dominance, outcompeting the wild type (WT; identical to Wuhan-Hu-1) and single mutants (described below). These dynamics could result from nonadditive, epistatic interactions among the mutated sites (10, 11) or simply from additive effects of multiple mutations (11). The effects of all single mutations in the RBD relative to the WT have been studied, and several mutations producing partial antibody escape have been identified (8, 12). Epistasis among RBD mutations cannot be characterized through the study of the WT alone, and pronounced epistasis within the RBD would make assessing the likelihood of vaccine escape for newly emergent variants of concern (VOC) extremely challenging. Moreover, epistatic interactions could result in systematic stabilization or destabilization of NAb-RBD or ACE2-RBD complexes for a variety of RBD mutations, potentially broadly promoting vaccine escape.

Using the Rosetta software suite, https://rosettacommons.org (13), we estimated and compared the effects of all single nonsynonymous mutants at the RBD-NAb and RBD-ACE2 interfaces for the WT as well as Delta (452R, 478K), Gamma (417T, 484K, 501Y), and Omicron (339D, 371L, 373P, 375F, 417N, 440K, 446S, 477N, 478K, 484A, 493R, 496S, 498R, 501Y, 505H) variants. The Delta and Gamma variants were dominant in different regions of the world with rising frequencies as of Summer 2021 (14, 15). Delta rose to global dominance in the following months, and at the time of writing, Omicron is rapidly growing in frequency and is expected to become the next globally dominant strain. We establish the distribution of RBD mutations on the plane bounded by the costs of ACE2 and NAb binding and classify the direction and magnitude of epistatic interactions between variant mutations and the broader mutational repertoire. The results reveal only weak epistasis, which, although more pronounced for the Gamma and Omicron variants than for the Delta variant, suggests limited potential for continued evolution toward more complete vaccine escape.

## RESULTS

### Rationale.

**FIG 1:**
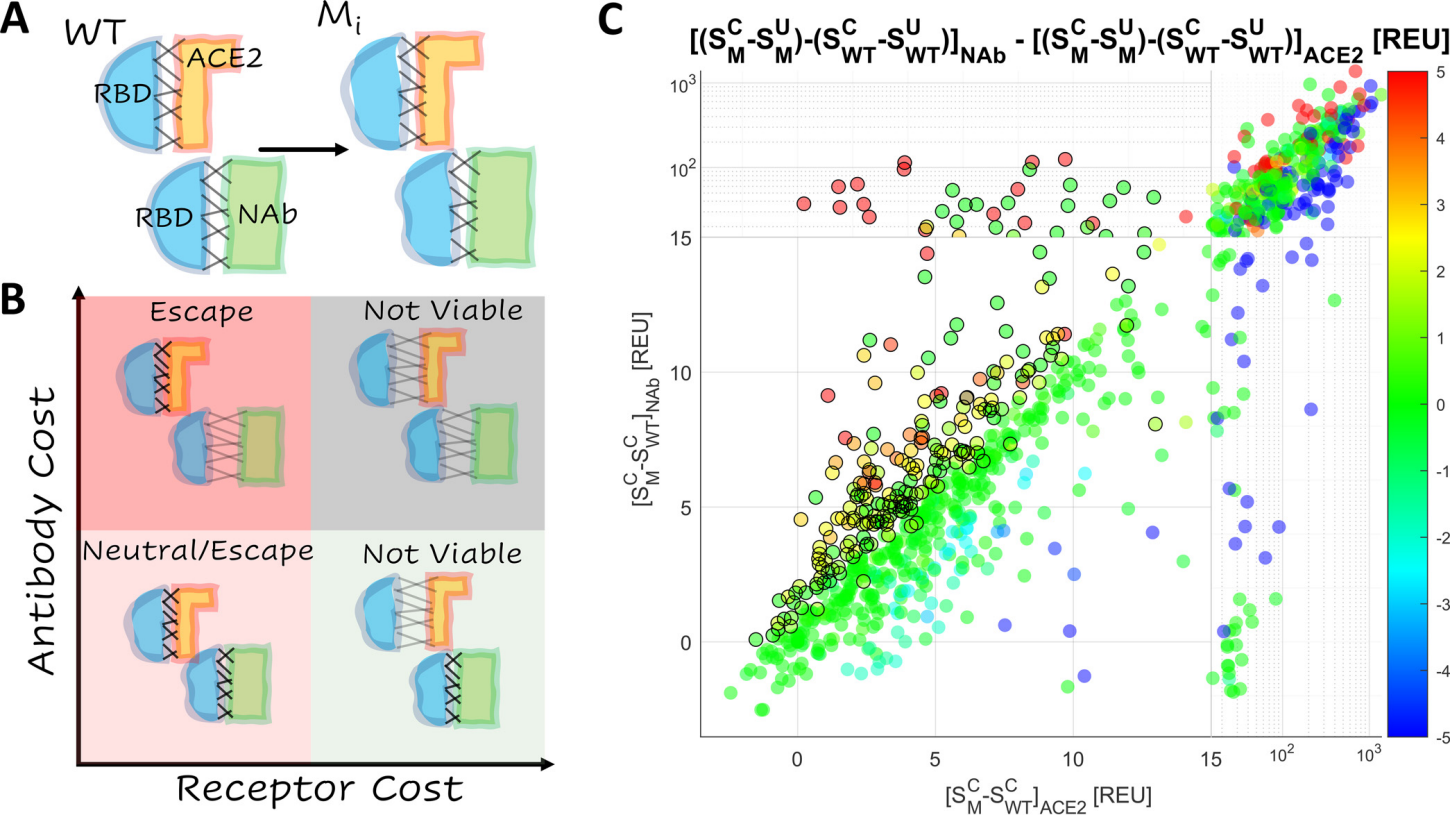
Landscape of vaccine escape mutants for the WT RBD. (A) Cartoon depicting unique conformational changes to the RBD (blue) in complex with ACE2 (orange) and the NAb (green) associated with the same mutation. (B) Cartoon depicting the landscape of vaccine escape mutations (the plane of receptor cost versus antibody cost). (C) Landscape of vaccine escape mutations for the WT RBD. Circles with a black outline are NAb escape candidates. Color indicates propensity for escape as measured by ΔΔG.

Epistatic interactions among mutations in the RBD of SARS-CoV-2 S protein are of interest and concern because they might substantially increase the risk of vaccine escape. Mutations in the RBD subtly change the shapes of the interfaces between RBD and ACE2 and between RBD and NAb (Fig. 1A). While the structure of the WT RBD-ACE2 interface is highly similar to that of the RBD-NAb interface (described below), a single mutation in the RBD can result in distinct shape changes in both interfaces. These changes can be depicted by the position of each mutant on the plane bounded by the receptor binding cost and the antibody cost (Fig. 1B). The cost is the increase (positive cost) or decrease (negative cost) in the ACE2 or NAb binding affinity relative to the WT. The four quadrants of this plane (Fig. 1B) represent four broad categories of mutations. Mutations in the top right quadrant are strongly destabilizing relative to both ACE2 and the NAb. The bottom right quadrant contains mutants that strongly destabilize the interaction with ACE2 but not with the NAb. Most mutants in these quadrants are not evolutionarily viable. Mutants in the bottom left quadrant stabilize or only weakly destabilize both interfaces. These mutations may or may not provide a selective advantage to the virus depending on the fraction of the host population that has been vaccinated or has recovered from prior infection. The top left quadrant contains mutations that strongly destabilize the interaction with NAb but not with ACE2 and therefore are most likely to admit vaccine escape.

### Single-mutant vaccine escape candidates for the wild-type RBD.

Starting with the two crystal structures of interest, the RBD in complex with ACE2, https://www.rcsb.org/structure/6M0J (16), and the RBD in complex with the NAb CV30, https://www.rcsb.org/structure/6XE1, we generated a representative ensemble of 50 native conformations per complex by following standard Rosetta protocols (see Materials and Methods and Discussion for details). Although NAb that bind epitopes that do not overlap the RBD have been identified (8), at the time of writing, the antibodies most critical for assessing the risk of vaccine escape appear to overlap the RBD (9) and are well represented by CV30. Regions important for antibody binding are known to overlap broadly among human coronaviruses (17). We then identified the RBD residues at the interface for each conformation and, in all conformations, introduced all single amino acid substitutions at these sites. For the WT and Delta variant, 52 residues (Table 1) were identified at the interface of at least one conformation for either complex. Four additional residues were identified for the Gamma and/or Omicron variants. Sites 480 and 488 are connected by a disulfide bond and were found to be unsuitable for substitution.

**TABLE 1:** Receptor-binding and antibody-binding interface footprints in the RBD

Footprint	Site(s)
WT RBD-ACE2	403, 405, 417, 445–447, 449, 453, 455–456, 473–478, 484–491, 493–498, 500–506
Delta RBD-ACE2	Same as WT
Gamma RBD-ACE2	WT + 404, 406, 408, 439, 499–484
Omicron RBD-ACE2	WT + 404, 439, 499–445, 484, 491
WT RBD-NAb	403-406, 409, 414–417, 419–421, 446–447, 449, 453, 455–461, 473–478, 484–498, 500–506
Delta RBD-NAb	Same as WT
Gamma RBD-NAb	WT + 408, 480
Omicron RBD-NAb	WT − 446

All 19 substitutions in each of the remaining 54 sites were investigated, with the exception of WT reversion for the variants. Note that substitutions made in the WT are all single mutants; those in the Delta variant are triple mutants (given that this variant contains two mutations in the RBD); those in the Gamma variant are quadruple mutants; and those in the Omicron variant each encompass 16 mutations in total. We chose to examine all variants containing Delta/Gamma/Omicron substitutions and an additional substitution at the interface rather than probing a randomly selected, representative ensemble of multiple mutants relative to the WT, because the mutations present in the Delta/Gamma/Omicron variants are known to be of biological and epidemiological relevance and the space of all such multiple mutants is prohibitively large. Altogether, this analysis produced more than 400,000 structures, which necessitated the development of a computationally efficient and, therefore, simplified protocol. To address this need, mutants were introduced into each conformation without repacking of adjacent sidechains or backbone minimization. This minimalist approach yielded favorable comparisons to the available experimental data (described below). However, generally, substitutions might introduce steric or charge clashes within the conformations in which the mutations were introduced (without repacking and minimization). Inference of the relative change in binding affinity for the ACE2 and NAb complexes is limited for such mutations. However, we observed a favorable comparison to experimental data in this respect as well, whereby few experimentally predicted escape mutations (relative to the WT) fall into this inference-limited category (described below).

Structure stability was estimated by the total score, *S*, in arbitrary units produced by the empirically driven Rosetta Energy Function 2015 (18) (labeled REU, for Rosetta energy units; https://new.rosettacommons.org/docs/latest/rosetta_basics/Units-in-Rosetta). The total score was calculated for each of the 50 conformations of the NAb and ACE2 complexes generated, and the mean value was assessed with, $S_{M}^{C}$, and without, $S_{\mathrm{WT}}^{C}$, the mutation. The receptor cost and antibody cost were estimated as ${\left[S_{M}^{C}-S_{\mathrm{WT}}^{C}\right]}_{\text{ACE2}}$ and ${\left[S_{M}^{C}-S_{\mathrm{WT}}^{C}\right]}_{\mathrm{NAb}}$ respectively. The interface free energy (ΔG) was also more directly approximated by the difference between the total score of the unbound state and the complex, $S^{C}-S^{U}$. The effect of the mutation on this value (ΔΔG) was reported for both complexes, $\left(S_{M}^{C}-S_{M}^{U}\right)-\left(S_{\mathrm{WT}}^{C}-S_{\mathrm{WT}}^{U}\right)$. Figure 1C shows the distribution of RBD mutations on the plane bounded by the ACE2 and NAb binding costs and putative NAb escape candidates, for which ${\left[S_{M}^{C}-S_{\mathrm{WT}}^{C}\right]}_{\mathrm{NAb}}-{\left[S_{M}^{C}-S_{\mathrm{WT}}^{C}\right]}_{\mathrm{ACE}2}>1$ or ΔΔG_NAb_ − ΔΔG_ACE2_>1 and ${\left[S_{M}^{C}-S_{\mathrm{WT}}^{C}\right]}_{\mathrm{ACE}2}<13$. The threshold value of 13 was selected to remove from consideration mutations that likely produce steric or charge clashes in the structure. Approximately one-third of all mutants studied lie above this threshold. As discussed further below, few viable mutations that reduce the neutralizing activity (and therefore represent escape candidates) of antibodies COV2-2050 and COV2-2479 in the WT (8) fall above this threshold. Moreover, radical amino acid substitutions that result in unresolved steric clashes are also likely to disrupt the RBD-ACE2 interface after backbone minimization, and we expect that the majority of mutations that are removed from consideration here for technical reasons are nonviable. Nonetheless, although not considered escape candidates, the potential for escape-exacerbating effects (greater NAb destabilization relative to ACE2 destabilization) of mutations above this threshold are assessed irrespective of the magnitude of ${\left[S_{M}^{C}-S_{\mathrm{WT}}^{C}\right]}_{\text{ACE2}}$, and interface sites without candidates but with a strong signature of escape exacerbation are discussed below.

Mutants showed strong clustering along the diagonal (identity line: receptor cost is equal to antibody cost), indicating that most mutations similarly affected the WT RBD-ACE2 and RBD-NAb complexes. Mutations in the top left quadrant of the plane, which corresponds to strong destabilization of the interaction with NAb but not with ACE2, are the strongest candidates for vaccine escape, followed by those in the bottom left quadrant, which includes weakly destabilizing mutations. The selective advantage (or lack thereof) of mutations in this quadrant depends on the fraction of the host population that has been vaccinated or has recovered from prior infection (see Discussion). In a fully vaccinated population, mutations that substantially reduce infectivity through the destabilization of receptor binding while also destabilizing the interaction with NAb could still provide a selective advantage. In particular, multiple mutations in 6 sites (417, 477, 484, 491, 493, and 499) were found to substantially destabilize the RBD-NAb complex relative to the RBD-ACE2 complex (see Fig. S1 in the supplemental material). Additionally, we identified site 453 to harbor mutations that simultaneously stabilize the RBD-ACE2 complex and destabilize the RBD-NAb complex. These observations are broadly consistent with the results of deep mutational scanning (8, 9, 19–22). Therefore, we conservatively considered all mutations for which the antibody cost exceeded the receptor cost to be viable escape candidates.

### Single-mutant vaccine escape candidates for the Gamma, Delta, and Omicron variant RBDs and predicted epistatic interactions.

**FIG 2:**
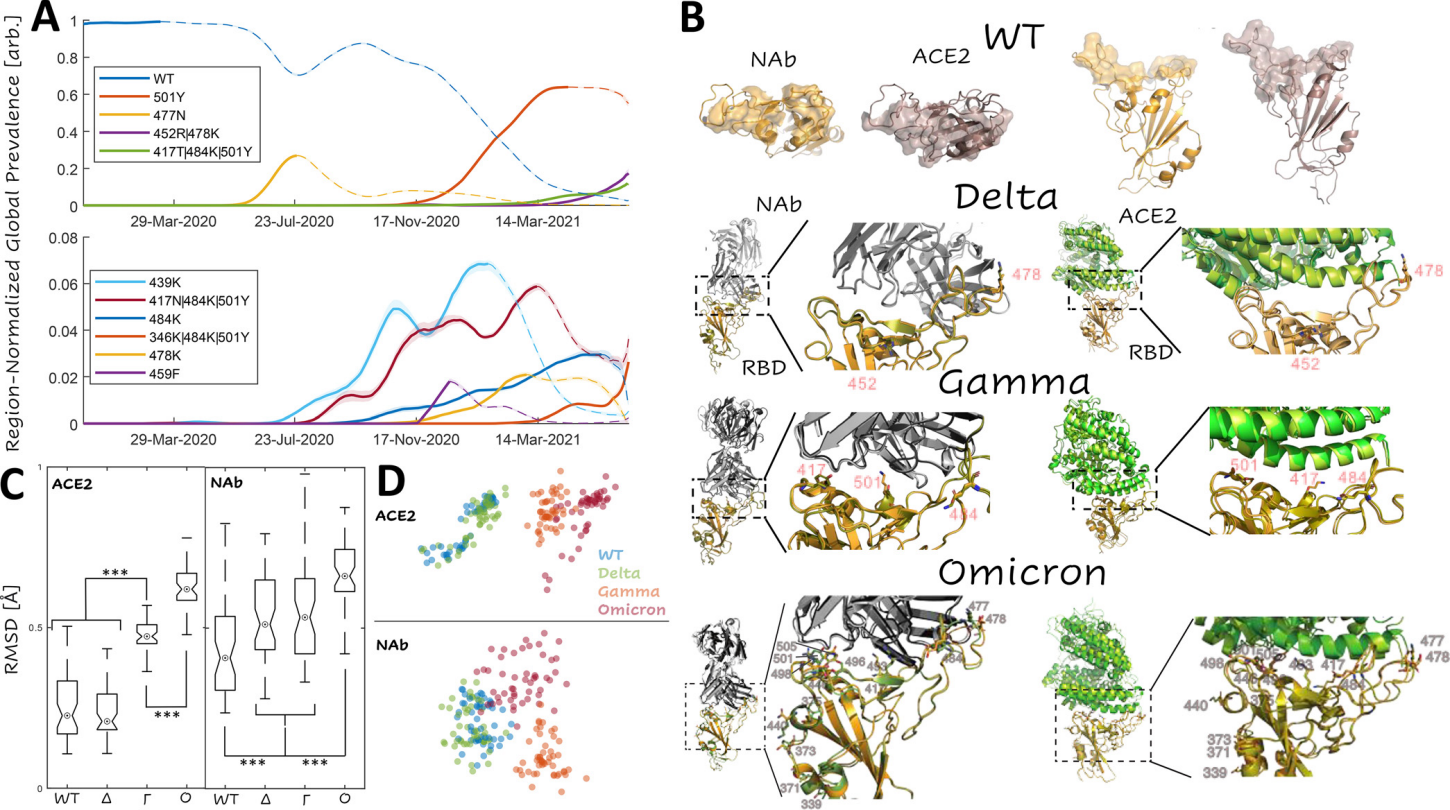
Dominant trends in circulating RBD mutations. (A) Region-normalized global prevalence of the top 10 most common combinations of RBD mutations over time. Lines are solid up to peak prevalence and dashed afterwards. Shading indicates confidence intervals. (B) Structural comparison of the complexes of ACE2 and NAb with RBD for WT, Delta, Gamma, and Omicron variants. (Top) WT footprints including the residues with an interaction within 4 Å of the partner. (Bottom) Visualization of the Delta, Gamma, and Omicron variant interfaces. Mutations are labeled and represented as sticks. WT structures are superimposed for the RBD of each variant: WT, orange; variant, olive. (C) Interface RMSD for ACE2 and NAb complexes relative to an arbitrary WT conformation (over spike protein sites 403 to 406, 408 to 409, 414 to 417, 419 to 421, 439, 445 to 447, 449, 453, 455 to 461, 473 to 478, 480, 484 to 506). Asterisks denote *P* values less than 0.02 for a Wilcoxon rank sum test. (D) CMDS applied to the pairwise RMSDs among all RBD-ACE2 and RBD-NAb complexes. Two-dimensional (2D) visualizations of 3D projections are displayed (see Fig. S2 for 3D).

Having charted the ACE2-binding and NAb-binding landscapes for the WT RBD, we sought to identify the most prominent combinations of RBD mutations circulating over the course of the pandemic. As of 16 June 2021, there were 53 countries from which more than 1,000 SARS-CoV-2 isolates were contributed to the GISAID (23) database (see “Data availability,” below). For each of these locations, we randomly selected 1,000 isolates and reported the frequency of each combination of RBD mutations among the 53,000 selected isolates over time. Figure 2A displays this region-normalized global prevalence of the 10 most common combinations of RBD mutations. The 6 RBD single mutants (501Y/Alpha variant, 477N, 439K, 484K, 478K, and 459F) began emerging between July and November 2020. All these single mutants were eventually displaced by 4 RBD multiple mutants (452R|478K/Delta variant, 417T|484K|501Y/Gamma variant, 417N|484K|501Y/Beta variant, and 346K|484K|501Y), which began emerging in November 2020, with the exception of Beta, which, according to our analysis, first appeared in July. By March 2021, the WT had become less prevalent than the Alpha, Gamma, and Delta variants. We pursued further analysis for the Delta, Gamma, and recently identified Omicron variant RBDs given their high and rising global prevalence. The complexes were prepared starting from the WT crystal structures and treated identically to the WT. We also discuss how similar results can be obtained, starting directly with the native conformations approximated for the WT, substantially reducing the computational burden (see Materials and Methods).

The rapid emergence and subsequent displacement of RBD single mutants might in part result from epistasis among the RBD variant residues or could be due to purely additive interactions. The most prominent trend was the displacement of the single mutant, 501Y (Alpha variant), by the Beta and Gamma variants (both also containing 501Y). Residue 501Y has been shown to substantially increase the binding affinity with ACE2, which, however, is reduced with the addition of mutation 417N in the Beta variant (11). In contrast, 417N severely reduces the neutralizing activity of a variety of NAb (24). These observations imply that mutations in site 417 provide a selective advantage through destabilization of the NAb complex, but given the large overlap between the RBD-NAb and RBD-ACE2 interfaces, maintenance of sufficient infectivity requires a compensatory mutation, such as 501Y, that stabilizes the RBD-ACE2 complex. Note that the emergence of these variants preceded widespread vaccination and that although the competition between antibody and receptor binding occurs even during an infection of a naive host, evolutionary pressures are likely to shift with increasing rates of vaccination and prior infection (described below). At the time of writing, the origin of the Omicron variant and, thus, the evolutionary pressures that led to its emergence remain unknown (see Discussion); however, despite bearing many more RBD mutations, the epistatic landscape at the interface is highly similar between the Gamma and Omicron variants (described below).

Examination of the interface footprints, defined as the ensemble of sites predicted to lie at the interface of at least one of the 50 conformations for each complex, for the 8 complexes of interest demonstrates that the RBD makes a greater number of contacts with NAb than with ACE2 within the same range of sites, 403 to 506 (Fig. 2B). The footprints of the WT and Delta variant interfaces in both the RBD-ACE2 and the RBD-NAb complexes are identical. The WT/Delta RBD-ACE2 footprint consists of 37 sites, whereas the WT/Delta RBD-NAb footprint includes 51 sites (Table 1). The Gamma RBD-ACE2 footprint consists of 41 sites, including all those in the WT/Delta footprint, with the single exception of site 484, and five additional sites. The Gamma RBD-NAb footprint consists of 53 sites, including all those in the WT/Delta interface and sites 408 and 480. The Omicron RBD-ACE2 footprint consists of 37 sites with three WT sites missing (including 484) and three additions. The Omicron RBD-NAb footprint includes 50 sites with one WT site missing.

Sites in the RBD-NAb footprint that are not shared by the RBD-ACE2 footprint might provide routes for the emergence of vaccine escape variants. However, because the RBD-ACE2 footprint is smaller than the RBD-NAb interface, the former is more sensitive to perturbation than the latter, for example, from mutations in site 417, which is part of the footprint of all 8 complexes. Notably, site 484 is absent from the Gamma and Omicron RBD-ACE2 footprints but remains in the respective RBD-NAb footprints. Also of note, site 446, which is mutated in Omicron, is present in the RBD-NAb footprint but not the RBD-ACE2 footprint for this variant. Consistent with the differences in the footprints, we found the Omicron and, to a lesser extent, Gamma variant RBD conformations in complex with ACE2 to be significantly different from that of the WT and Delta variants, which could not be differentiated from one another. All variant RBD conformations in complex with the NAb were found to be significantly different from that of the WT. For Delta and Gamma, this difference was modest and smaller in magnitude than the variability among the RBD-NAb conformations, whereas Omicron showed a more pronounced difference from the WT (Fig. 2C).

Classical multidimensional scaling (CMDS) applied to the pairwise interface root mean square distances (RMSDs) among all RBD-ACE2 and RBD-NAb complexes showed that the Gamma and Omicron RBD-ACE2 structures lie on a shared continuum of conformational change relative to the WT, with the Omicron conformations being closer to Gamma than to the WT. In contrast, Omicron, Gamma, and, to a lesser extent, Delta RBD-NAb structures all represent distinct conformational changes relative to the WT (Fig. 2D and Fig. S2). Despite these differences, the effects of mutations in the RBD were found to be principally additive in all variants, that is, there seems to be little epistasis.

**FIG 3:**
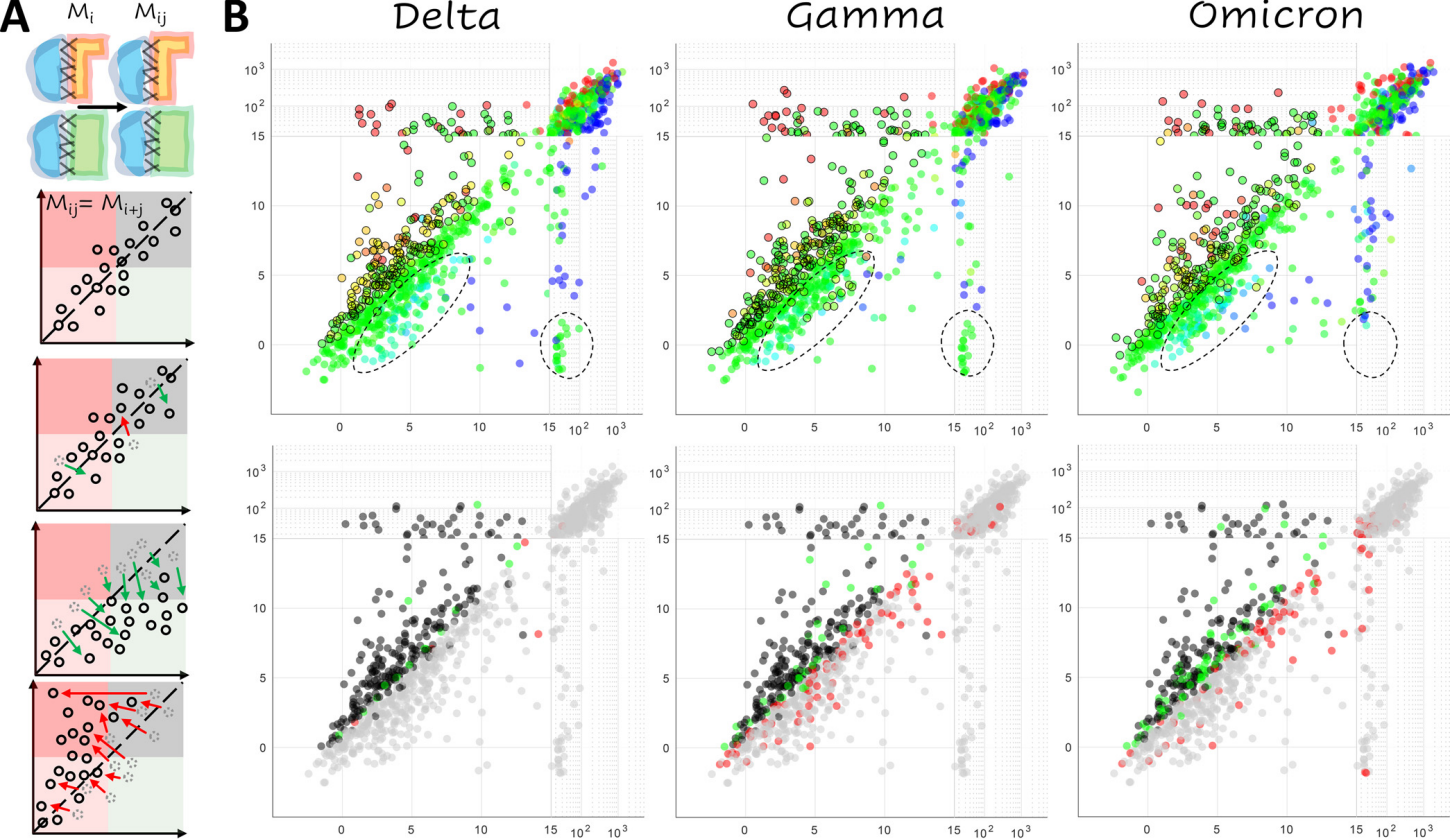
Epistasis within the RBD. (A) Cartoon illustrating additive and nonadditive (epistatic) interactions between mutations. From top to bottom are additive, escape-neutral, escape-minimizing, and escape-exacerbating mutations. *Mi*, *Mj*, and *Mij* denote the effects of the single and double mutants. (B, top) Landscape of vaccine escape mutations for the variant RBDs. Coloring, as in Fig. 1C, indicates propensity for escape as measured by ΔΔG. Circles with a black outline denote NAb escape candidates. Dashed lines highlight differences among variants. (Bottom) Landscape of vaccine escape mutations for the WT RBD. Black points are candidates for both the WT and variant; gray points are not candidates for either the WT or variant; green points are only candidates for the WT; red points are only candidates for the variant.

When a second mutation, *M_j_*, is introduced in addition to a prior mutation, *M_i_* (Fig. 3A), the resulting conformational change can be additive so that the effect of the two mutations is the sum of the effects of the two individual mutations. In this case, the position of the double mutant *M_i__j_* on the plane defined by the receptor cost and antibody cost relative to the single mutant, *M_i_*, will be the same as that of the single mutant, *M_j_*, relative to the WT. If the conformational change is nonadditive, representing an epistatic relationship, the resulting trends can be classified by their impact on potential vaccine escape. Such trends could be escape neutral when the ensemble of candidate vaccine escape mutations differs from that for the WT but the number of such candidates is the same; escape minimizing when the antibody cost is, on average, reduced relative to the receptor cost across all mutations for the mutant versus the WT; or escape exacerbating where the antibody cost is, on average, increased.

The landscape of mutants predicted to enhance vaccine escape for the Delta variant was almost identical to that of the WT but differed significantly from the Gamma and Omicron landscapes (Fig. 3B). These trends are summarized in Table 2, which tabulates all nonshared candidates. There are 15 (13) escape candidates in the WT that were not predicted to enhance escape for Delta and 6 (2) candidates in Delta but not WT (values in parentheses are mutations with ${\left[S_{M}^{C}-S_{\mathrm{WT}}^{C}\right]}_{\text{ACE}2}<13$, regardless of whether or not the mutation is a candidate, a threshold chosen to mitigate potential artifacts caused by steric or charge clashes). In contrast, in the case of Gamma, there were 32 (28) candidates identified in the WT but not Gamma and 86 (66) candidates identified in Gamma but not the WT. Omicron demonstrated intermediate behavior with 67 (59) candidates identified in WT but not Omicron and 75 (48) candidates identified in Omicron but not the WT. Thus, we identified 9 (11) fewer escape candidates for Delta than the WT but 54 (38) additional candidates for Gamma and either 8 additional candidates (ignoring potential steric/charge clashes in the WT) or 11 fewer candidates for Omicron.

**TABLE 2:** Numbers of antibody escape candidates^*a*^

Virus	No. of candidates	Greek letter	No. for WT not variant	No. for variant not WT	Difference
WT	241		15 (13)	6 (2)	−9 (−11)
Delta	232	Δ	32 (28)	86 (66)	54 (38)
Gamma	295	Γ	67 (59)	75 (48)	8 (−11)
Omicron	249	Ο			

a Values in parentheses are mutations tabulated with ${\left[S_{M}^{C}-S_{\mathrm{WT}}^{C}\right]}_{\text{ACE2}}<13$ for both the WT and variant.

Consistent with the more dramatic conformational change observed in the RBD-ACE2 complex relative to the RBD-NAb complex, the nonadditive effects observed in the Gamma variant appear to predominantly result from the decreased sensitivity of the RBD-ACE2 interface to mutation. This conclusion is compatible with the available experimental results. Figure S3 shows the distribution of the receptor cost, ${\left[S_{M}^{C}-S_{\mathrm{WT}}^{C}\right]}_{\text{ACE}2}$, for two categories of mutations (for each of the three receptor complexes): those at the interface that have been experimentally demonstrated to reduce neutralizing activity of antibodies COV2-2050 and COV2-2479 in the WT (8) and all others (included in the same experimental study) at the interface. As discussed above, the upper bound for the receptor cost, ${\left[S_{M}^{C}-S_{\mathrm{WT}}^{C}\right]}_{\text{ACE}2}$, is lower for mutations predicted to reduce NAb activity than for other mutations. However, the Gamma candidate ensemble exhibits a reduced median receptor cost. In other words, in the Gamma variant, mutations that are predicted to reduce NAb activity are also less likely than other mutations to reduce the receptor binding affinity relative to the WT and Delta variant. The three residues within the RBD-ACE2 footprint of the Omicron variant not present in the WT overlap those of the Gamma variant (404, 439, and 499), and although not statistically significant, the decreased cost of receptor binding was observed for Omicron as well. The Omicron variant additionally displayed a modest reduction in median receptor cost for the broader category of experimentally studied mutations, suggesting greater flexibility at the receptor interface (Fig. S3).

**FIG 4:**
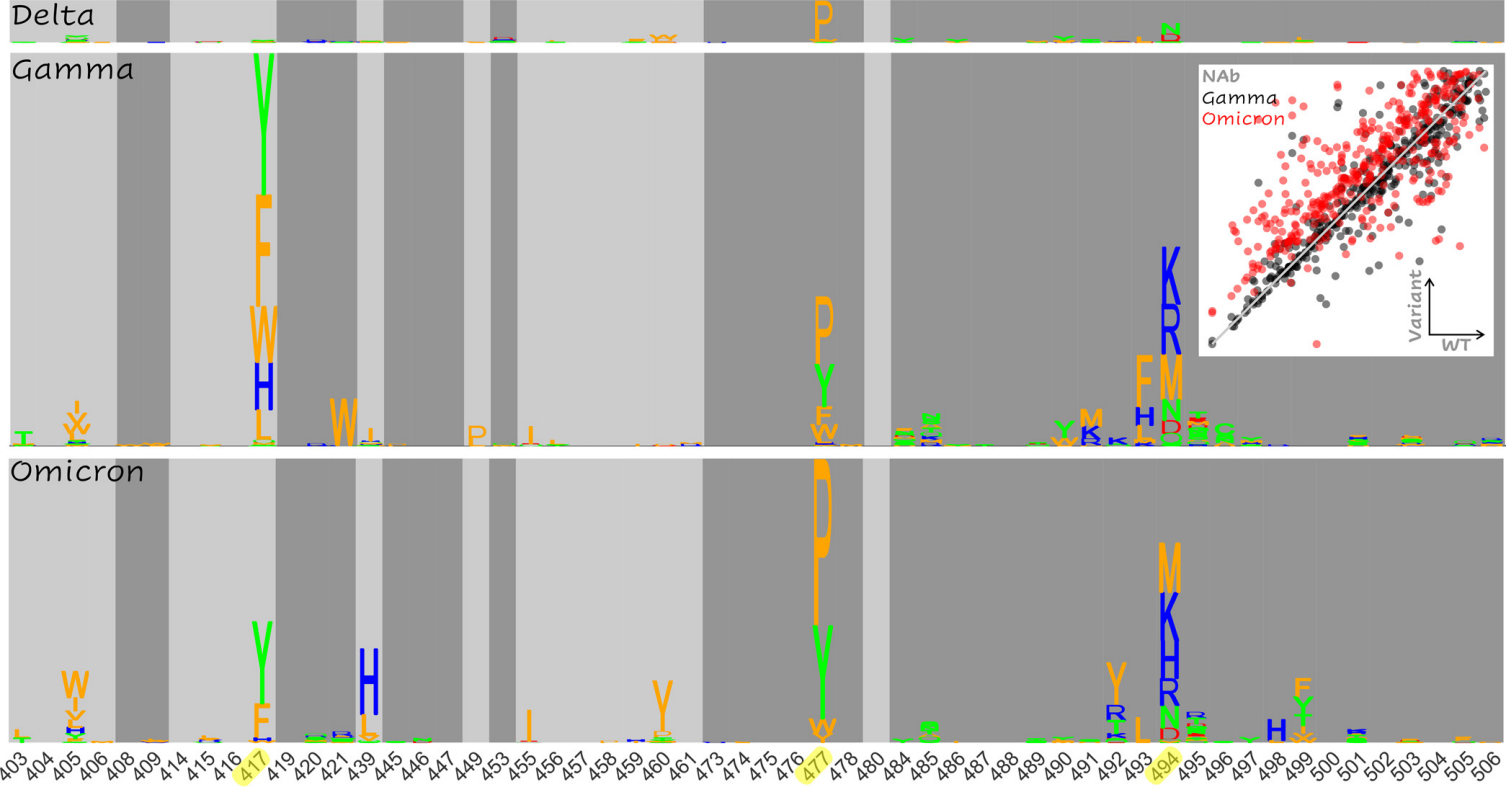
Landscape of epistatic effects supporting enhanced vaccine escape. Nonadditive escape-exacerbating motifs in Delta (top), Gamma (middle), and Omicron (bottom) variants are shown. The size of each letter corresponds to the increased likelihood of vaccine escape for the substitution in the variant relative to the WT. (Inset) Total score change (${\left[S_{M}^{C}-S_{\text{WT}}^{C}\right]}_{\text{NAb}}$) induced by mutation in the Gamma (black) and Omicron variants (red) versus the WT (identity line overlaid). The minimum value (minus 1) is subtracted from each distribution for normalization (see Fig. S5 for more detail).

Figure 4 summarizes the magnitude of the increased risk of vaccine escape for each mutation at the RBD interface (given ${\left[S_{M}^{C}-S_{\mathrm{WT}}^{C}\right]}_{\text{ACE2}}<13$) for each variant relative to the WT. Epistasis increasing the risk of vaccine escape is primarily apparent in three regions of the RBD interface, site 417, site 477, and site 494, together with the surrounding neighborhood. The trend in site 417 was observed only in the Gamma and Omicron variants, which already contain mutations at that site, showing that further changes to this site could result in enhanced vaccine escape. However, the epidemiological implications of this finding are limited considering that mutations in site 417 are likely to pose a risk of vaccine escape in most variants. The enhanced escape associated with mutations in site 477 for all variants relative to the WT, together with the early spread of 477N and the presence of 477N in the Omicron variant, suggest that this site plays an important role in host adaptation. Most prominently, mutations in site 494 and the surrounding neighborhood are likely to enhance vaccine escape in all variants. Indeed, 494P has been found in circulation and experimentally demonstrated to reduce antibody neutralization capacity of convalescent-phase sera (25).

In addition to these apparent differences among the ensembles of candidate vaccine escape mutations, we observed sites that harbored no candidates but nevertheless displayed signatures of increased risk of vaccine escape for Gamma and/or Omicron. The two most notable trends were observed in sites 408 and 504 (Fig. S4). All but one substitution in site 408 enhance vaccine escape for Gamma and Omicron, but surprisingly, all have the opposite effect in Delta. Similarly, all substitutions at site 504 substantially enhance vaccine escape in Gamma and Omicron but exert a modest opposite effect in Delta. However, these mutations are not considered candidates in our analysis because, even for Gamma and Omicron, they destabilize the ACE2 interaction to a greater extent than the interaction with NAb. Additionally, ${\left[S_{M}^{C}-S_{\mathrm{WT}}^{C}\right]}_{\text{ACE}2}<13$ for substitutions at site 504, limiting confidence in the assessment of trends at this site.

Importantly, few differences were observed between the epistatic landscapes of the Gamma and Omicron variants, mainly in sites 417, 439, 499 (Fig. 4), and 446 (Fig. S4). Sites 417 (reduced escape-related epistasis) and 446 (increased escape-related epistasis) are already mutated in Omicron, and the signatures in sites 439 and 499 are modest relative to the consistent trend observed in site 494. Nonetheless, it is important to acknowledge a small, systematic bias toward NAb destabilization within the Omicron variant that is not observed within Gamma or Delta (Fig. 4, inset, and Fig. S5). As demonstrated above, unlike the RBD-ACE2 complex, which displays a shared continuum of conformational change relative to the WT, RBD-NAb structures all represent distinct conformational changes (Fig. 2D and Fig. S2), with the greatest change observed for Omicron. Most mutations, which modestly destabilize the WT RBD-NAb complex (${\left[S_{M}^{C}-S_{\mathrm{WT}}^{C}\right]}_{\mathrm{NAb}}<10$), are slightly more destabilizing in the Omicron RBD-NAb complex than the other variants. As discussed above, only a subset of these mutations are considered candidates for antibody escape, which depends on the properties of the mutation within the RBD-ACE2 complex as well. The principal escape signatures are conserved between the Gamma and Omicron variants, with fewer escape candidates identified overall for the Omicron variant. However, this systematic destabilization of the Omicron RBD-NAb complex suggests, in principle, additional avenues toward vaccine escape.

## DISCUSSION

Here, we report the results of a computational study predicting the effects of all single mutants at the RBD-NAb and RBD-ACE2 interfaces for the WT as well as the Delta, Gamma, and Omicron variants of SARS-CoV-2 on receptor and antibody binding. For the WT, we found multiple mutations in 6 sites (417, 477, 484, 491, 493, and 499) that are predicted to significantly destabilize the RBD-NAb complex relative to the RBD-ACE2 complex and appear to pose a risk of vaccine escape, which is broadly consistent with the results of deep mutational scanning (8, 9, 19–22). Overall, most mutations at the interface were found to similarly affect the WT and all variants, indicating limited epistasis at the interface. Nonadditive, epistatic interactions predicted to increase the risk of vaccine escape were apparent, however, at sites 477 and 494 as well as in the surrounding neighborhood. This trend is particularly prominent in the Gamma variant so that, across all sites at the interface, we predicted 22% more escape candidate mutations for the Gamma variant than for the WT. In contrast, there is little apparent epistasis in the Delta variant, and across all sites at the interface, we predicted 4% fewer candidate mutations than WT. Despite harboring many more RBD mutations and displaying a small, systematic trend toward NAb complex destabilization, the Omicron variant demonstrated intermediate behavior, with only 3% more candidate escape mutations than the WT.

Epistasis is a major, if not the principal, driver of protein evolution (26). Compensatory mutations are particularly strong epistatic interactions that can result in chemotherapeutic (27) or antimicrobial (28) drug resistance and are commonly observed throughout species evolution (29). In a completely susceptible population, mutation 501Y, which appears to substantially increase infectivity (11), is expected to evolve under positive selection. As a population gains immunity through prior exposure and/or vaccination, selective pressures rapidly change to promote the emergence of resistant variants (7). Under these conditions, 501Y and other mutations, which increase infectivity, might primarily play the role of compensators for mutations destabilizing NAb interactions, such as 417N or 417T. As global vaccinations rise due to changing evolutionary pressures, it can be expected that more mutations emerge that destabilize the interactions of the RBD with both NAb and ACE2, resulting in (partial) escape variants that, however, also have reduced infectivity. However, variants such as Gamma and Omicron that carry both an antibody destabilizing mutation and a compensatory mutation have the potential to undercut this trend. This may further complicate the relationships between transmissibility, antibody neutralization, and pathogenicity, as potentially evidenced by the Omicron variant, with early reports suggesting increased transmissibility but decreased pathogenicity (30).

At the time of writing, the origins of the Omicron variant are unclear and it is suspected to have evolved over an extended period of time within an immunocompromised individual(s) or an animal reservoir (31). Such environments could present selective pressures distinct from the broader human host population, but the changing competition between receptor and antibody binding described above is likely to be conserved. Despite the more pronounced conformational change, the average behavior of the conformational ensemble selected to represent the Omicron variant was found to be less destabilizing for the receptor than Gamma but highly destabilizing for the antibody, indicating that our model for Omicron is at least as accurate, if not more accurate, than those for the Gamma and Delta variants (see Fig. S12 and S13 in the supplemental material).

Above all else, we find it important to highlight three points. (i) The epistatic landscapes of the Gamma and Omicron variants are highly similar (Fig. 4). (ii) We find fewer, not more, escape-exacerbating mutations in Omicron than Gamma (Table 2). (iii) These features persist despite RBD-ACE2 structures representing a shared continuum of conformational change relative to the WT, with the Omicron conformations being closer to Gamma than the WT (Fig. 2D and Fig. S2), indicating that the magnitude of conformational change does not trivially correlate with the propensity for exacerbated antibody escape. The neutralizing activity of existing vaccine-elicited NAb against the Omicron variant is likely to be substantially reduced (32), but multidose vaccination is expected to recover efficacy (33). Our results emphasize that although the adaptive repertoire of SARS-CoV-2 may be robust, structural constraints on the RBD make continued evolution toward more complete vaccine escape unlikely, suggesting continued efficacy of the existing vaccines.

The work presented here is strictly computational, and although we demonstrate agreement with experimental results where possible, many features not captured by the models presented (involving protein expression, docking, and other factors) could modulate antigen-receptor and/or antigen-antibody binding. Furthermore, although we explore many conformations for both the RBD-ACE2 and RBD-NAb interfaces, we start from a single crystal structure for each. We believe the conformational ensembles selected to represent each complex are diverse enough to accurately reflect the relative destabilization of the NAb and ACE2 complexes across the spectrum of RBD interface mutations, which is the primary concern. However, this conformational diversity makes it difficult to demonstrate stabilizing interactions, which are typically much weaker than destabilizing ones (12). Although multiple low-energy conformations were resolved for all variants and the WT, the average behavior of the conformational ensemble selected to represent the Delta variant relative to the WT was found to be weakly destabilizing for NAb and neutral for ACE2, whereas in the case of Gamma it was weakly destabilizing for both complexes. This is unlikely to accurately reflect the relative binding affinities between these variants and the WT given the enhanced infectivity of both variants, particularly Delta (14). However, it should be recognized that the relationship between the measures of interface stability we report and viral life history traits (infectivity, immune activity, etc.) is complex.

Although we believe we proposed sensible thresholds for determining which structures can be analyzed with high confidence and the biological implications of the relative destabilization of the NAb versus ACE2 are overt, the effects studied in this work do not represent the entire diversity of possible host adaptation. It is incompletely understood at the time of writing why the Delta variant appears to replicate faster than the WT (14), and substitutions outside the Spike protein could play key roles in immune modulation (34, 35). Even less is known about the Omicron variant. A targeted exploration of the lowest-energy conformations achievable for each variant might yield better agreement with the known properties of these variants and, in particular, reveal stabilizing RBD-ACE2 interactions. However, this would likely come at the cost of generalizability and decrease the power of our approach to predict relative destabilization of interface mutations between NAb and ACE2 complexes.

We also emphasize that we limit our study to the RBD of the S protein. In principle, epitopes located outside the RBD and far from the interface could play an important role in the emergence of vaccine escape variants by decreasing the similarity between the interaction of the antigen with the receptor and the interaction between the antigen and an antibody (36). However, if such effects were dominant, the satisfactory agreement between our predictions, deep mutational scanning, and the observed frequency of mutations within the RBD among the circulating variants presumably would not be recovered. Nevertheless, many routes of adaptive evolution are potentially available to this virus so that agreement with prior results is not a guarantee of predictive success. Although we believe our results strongly suggest a limited repertoire of escape-mediating mutations within the RBD, the possibility should be considered that mutations outside the RBD have the potential to increase this repertoire. In particular, important evolutionary relationships appear to exist between mutations within the S and N proteins (3). Such distant interactions might represent substantial, perhaps compensatory, interactions but are less amenable to the quantitative assessment of nonadditivity using the approach presented here. At intermediate distances, interactions between mutations within the S protein inside and outside the RBD, particularly in the N-terminal domain (NTD), could alter the antibody binding landscape (36). These relationships will have to be clarified in subsequent studies using enhanced computational and experimental approaches.

Finally, we note that the space available for neutral evolution, even within the RBD alone, is large. In principle, this enables acquisition of many RBD mutations, some combinations of which might exhibit substantially greater escape-exacerbating epistatic effects than the variant substitutions explored in this work. However, this appears unlikely considering the large number of mutations (15) observed in the Omicron variant. We believe this variant is an excellent test case to probe the limits of epistatic potential at the RBD interface.

### Conclusions.

We employed a computational approach to study the effects of all single mutations at the RBD-NAb and RBD-ACE2 interfaces for the WT as well as the Delta, Gamma, and Omicron variants of SARS-CoV-2. Overall, little epistasis at the RBD interface was detected, with additive effects on the binding affinities observed for most pairs of mutations. In the Delta variant, the observed nonadditive trends weakly stabilize the interaction of the RBD with the NAb relative to the interaction with ACE2, whereas in the Gamma variant, epistasis is predicted to more substantially destabilize interaction with the NAb relative to ACE2. The predicted epistatic landscape of the Omicron variant closely resembles that of Gamma, with an additional small systematic bias toward NAb destabilization but with fewer predicted escape candidates overall. In summary, while the Omicron variant poses new risks not observed for Delta, including the evolution toward greater NAb destabilization, structural constraints on the RBD make the continued evolution toward more complete antibody escape unlikely. Multiple variants have already demonstrated antibody evasion, and there is a high likelihood that new variants of concern will continue to emerge. However, the results presented here suggest that these variants will largely be defined by a modest ensemble of mutations already identified and that the effects of introducing a new mutation in such variants will be similar to the effects of introducing that mutation into the WT. Such propitiously boring epistasis substantially limits the repertoire of possible future escape mutations and predicts continued efficacy for the existing vaccines.

## MATERIALS AND METHODS

### Selection of crystal structures.

In this work, we considered a single crystal structure of the SARS-CoV-2 spike protein RBD in complex with the human receptor, angiotensin converting enzyme 2 (ACE2), PDB entry 6M0J (16). While there are likely multiple mutations outside the RBD that significantly affect binding characteristics of the spike protein, as has been demonstrated for site 614 (37), the structure of the RBD itself is unlikely to be substantially modified by such mutations. Thus, while only able to reveal a subset of mutations of interest, the focused study of RBD complexes presented here remains biologically realistic.

Similarly, we consider a single crystal structure of the RBD in complex with a neutralizing antibody (NAb), CV30, PDB entry 6XE1 (38). CV30 was recognized early on as the most potent NAb observed within the sera of a SARS-CoV-2-positive donor, while the majority of antibodies were found to target nonneutralizing epitopes outside the RBD (39). Subsequently, it became clear that antibodies targeting epitopes outside the RBD, particularly those targeting the N-terminal domain (NTD), likely play a protective role, and mutations reducing the affinity of these antibodies may be epidemiologically significant (36, 40). As acknowledged above regarding the selection of the spike crystal structure, while our focus on CV30 can only reveal a subset of mutations of interest, the mutations discussed in this work predicted to affect CV30 binding affinity are likely highly epidemiologically relevant.

### Construction of representative ensembles of interface conformations.

Protein crystal structures may differ substantially from the native conformations (41). Throughout this work, we utilize the Rosetta (13) software suite to approximate both wild-type (WT) and mutant conformations of the receptor and NAb complexes. All protocols used throughout were implemented using the RosettaScripts (42) package, and the XML files used along with the associated executed command lines are available in Table S1 in the supplemental material. Approximation of the native conformational ensemble may be separated into two steps, identifying the optimal side chain conformation (repacking) and moving the protein backbone (minimization), to minimize the energy function applied. This can be accomplished using the Rosetta Relax application, which iteratively applies each of these two steps.

Beginning with the crystal structure, we iteratively applied the FastRelaxMover using default parameters (with the exception of disabling design) for up to 12 iterations (15 for Omicron) and up to 1,000 repeats. We found the total score to be insensitive to additional applications of FastRelax after 5 iterations, on average. Each resulting structure was scored using the InterfaceAnalyzerMover, repacking the unbound state but not the bound state (as the input complex has already been optimized).

This protocol returns the total score, *S*, in arbitrary units produced by the empirically driven Rosetta Energy Function 2015 (18) (labeled REU, for Rosetta energy units, https://new.rosettacommons.org/docs/latest/rosetta_basics/Units-in-Rosetta) as well as dG separated, which is the difference in the total score between the bound (complex) and unbound state, *S^C^ − S^U^*, derived from separating the binding partners. This protocol can also be used to identify the residues within the complex that constitute the interface.

Such an ensemble of structures often forms an “energy funnel” (43) where the root mean square distance (RMSD) between superimposed backbone carbon atoms of each structure and the structure with the lowest total score or the lowest dG separated is positively correlated with the total score of that structure. To evaluate whether such a funnel exists for these ensembles, we identified the structure with the lowest dG separated and 90 residues predicted to be interface residues within the RBD in at least one conformation in addition to the ±3 adjacent amino acid neighborhood of each such residue (sites 400 to 424, 440 to 464, and 470 to 509 in the spike protein for WT; additional sites 436 to 439 for Delta; additional sites 434 to 439 for Gamma; and additional sites 434 to 439 and 510 for Omicron).

Figure S6 displays dG separated versus the interface RMSD for both complexes for the WT. Few structures appear in the lower left corner of each plot from only one or two (NAb or ACE2, respectively) independent “trajectories” of iterative FastRelax application, beginning with the crystal structure. Furthermore, while the lowest dG separated is more than 30% greater in magnitude than the highest for both complexes, the interface RMSD between any complex and the minimum dG separated complex is less than an angstrom. These findings suggest selecting the single minimum dG separated conformation for either complex is unlikely to constitute a realistic model of the native interface and may in fact represent an unrealistic, entropically disfavored state (44).

Figures S7 to S9 display dG separated versus the interface RMSD for both complexes for the Delta, Gamma, and Omicron variants. The distribution of conformations for the NAb is similar to that of the WT for both Delta and Gamma; however, the minimum dG separated obtained for Gamma is higher than that for the WT, suggestive of antibody destabilization for this variant. dG separated for Omicron is substantially higher, indicating significant destabilization. For Delta and Omicron, the distribution of conformations for ACE2 more resembles a funnel suggestive of receptor stabilization for these variants. For Gamma, there appear to be 2 to 3 distinct, equally low-energy conformations for ACE2, which makes the interpretation of the energy landscape more challenging. For consistency, representative conformations were selected according to the same protocol for all variants.

The construction of an ensemble of representative conformations is desired so that the average behavior of such an ensemble is likely to reflect that of the native complex. Ideally all available structures would be statistically weighted and included in this ensemble; however, this is computationally intractable. Instead, we selected 50 conformations for each complex as follows (Fig. S10 to S13). (i) The conformation corresponding to the minimum total score after any iteration of FastRelax from each independent trajectory beginning with the crystal structure was selected. (ii) The 10 structures with the lowest total score were removed, and the 10 structures with the lowest dG separated were removed (these are not identical structures and, as discussed above, may represent unrealistic, entropically disfavored conformations). (iii) The remaining structures were ranked by total score, *r_S_*, and dG separated, *r_G_*, and the 50 structures with the lowest composite rank, *r_S_ + r_G_*, were selected. The PDB files for these structures are available (see “Data availability,” below). We believe the equally weighted average behavior of these conformations, which are stable with well-resolved interfaces, constitute a reasonable model of the native complexes. Note that while we do not expect the results presented to substantially change if the lowest energy conformation out of this ensemble was selected for each mutation individually, as discussed above, selecting a single conformation to represent all mutations is likely to substantially reduce the generalizability of this approach.

### Analysis of single mutants relative to WT, Delta, and Gamma.

Given the 100 structures selected as described above (50 for each complex), a more restrictive list of 52 residues were predicted to lie at the interface of at least one structure for WT/Delta (spike protein sites 403 to 406, 409, 414 to 417, 419 to 421, 445 to 447, 449, 453, 455 to 461, 473 to 478, 484 to 498, 500 to 506), 56 for Gamma (WT/Delta + 408, 439, 480, 499), and 53 for Omicron (WT/Delta + 439, 499 to 445). We introduced all of the 19 possible mutations at each interface site (all 56 identified for any variant) for these 100 structures for the WT, Delta, Gamma, and Omicron variants, with the exception of WT reversion for the variants. Mutations were introduced using the PackRotamersMover with design specified by the input resfile (see “Data availability,” below). Only the targeted residue was modified; side chain conformations for all other residues were fixed, and no backbone minimization was applied before filtering for candidates of interest, as described below.

Introducing the mutation in this way without repacking and minimization results in many unrealistic, high-energy conformations that are difficult or impossible to interpret without further optimization. The benefit is speed. This procedure can be executed in under 30 s per structure, while global repacking and minimization may take hours. An alternative approach is to apply backbone minimization and side chain repacking within a local region of the structure centered at the modified site as implemented within Rosetta through the Flex ddG protocol (45). This approach has been used to accurately predict mutations conferring increased infectivity for SARS-CoV-2 (46) and rationally design NAb against the antigen (47, 48) but is significantly slower than introducing mutations without even local optimization. With or without local optimization prior to filtering, global repacking and minimization may be desired to confirm top candidates. While our approach may introduce a higher false-negative rate than what one would achieve by applying local optimization prior to filtering, it maximizes the breadth of candidate mutations considered and can recapitulate experimental results. Furthermore, it is important to note that even without repacking of adjacent side chains, the optimal rotamer was selected for the new amino acid introduced at the mutated site.

The total score and dG separated of the mutant structures were computed. Unlike in earlier steps where the unbound state was repacked during the dG separated calculation, no repacking was conducted for the reasons described above. Each mutant structure was matched with its initial WT/variant conformation, and the changes in dG separated, ($S_{M}^{C}$ − $S_{M}^{U}$) − ($S_{\mathrm{WT}}^{C}$ − $S_{\mathrm{WT}}^{U}$), and total score, $S_{M}^{C}$ − $S_{\mathrm{WT}}^{C}$, were calculated. The average of each value taken over the ensemble of the 50 RBD-ACE2 conformations was compared with experimentally determined ACE2 binding affinity for single RBD mutants (12). While the change in dG separated is technically the ddG for the mutant and may be expected to correspond to binding affinity, we find ($S_{M}^{C}$ − $S_{M}^{U}$) − ($S_{\mathrm{WT}}^{C}$ − $S_{\mathrm{WT}}^{U}$) over this ensemble is not generally predictive of ACE2 affinity, as ($S_{M}^{C}$ − $S_{M}^{U}$) − ($S_{\mathrm{WT}}^{C}$ − $S_{\mathrm{WT}}^{U}$) is approximately zero for many mutants (Fig. S14). This can be expected without repacking and minimization. Nonetheless, when nonzero, the change in dG separated compares favorably with ACE2 affinity (described below).

The change in total score is less sensitive, and an adequate predictor of affinity (Fig. S15) within the range of $S_{M}^{C}$ − $S_{\mathrm{WT}}^{C}$ < 5, such that the minimum relative binding affinity decreases with increasing change in total score. Additional validation that $S_{M}^{C}$ − $S_{\mathrm{WT}}^{C}$ is a reasonable predictor of binding affinity, below some threshold, can be obtained through demonstrating $S_{M}^{C}$ − $S_{\mathrm{WT}}^{C}$ for each mutant is highly correlated between the RBD-ACE2 complex and the RBD-NAb complex. Furthermore, when the difference between the change in dG separated for the NAb complex and the dG separated for the ACE2 complex, [($S_{M}^{C}$ − $S_{M}^{U}$) − ($S_{\mathrm{WT}}^{C}$ − $S_{\mathrm{WT}}^{U}$)]_NAb_ *−* [($S_{M}^{C}$ − $S_{M}^{U}$) − ($S_{\mathrm{WT}}^{C}$ − $S_{\mathrm{WT}}^{U}$)]_ACE2_, is positive, mutants lie above the identity line (*y* = *x*). The reverse is true for mutants falling below the identity line (Fig. 1C and 3B). In other words, on average, mutants in the RBD-ACE2 complex behave similarly to mutants in the RBD-NAb complex (which is what one would expect for realistic models of interfaces with an overlapping footprint). When differences do appear, they correspond to interactions with the RBD that are specific to the binding partner (ACE2/NAb) and are correctly reflected by the change in dG separated.

### Directly assessing additive effects versus epistasis.

In the main text, we demonstrate that most mutations appear in a similar position of the receptor cost/antibody cost phase space for the WT and Gamma/Delta variants. Consequently, the ensemble of vaccine escape candidates is largely conserved. Whether epistatic effects due to multiple mutations are present or if the impact of multiple mutations is simply additive can be more directly assessed for each complex by plotting the change in the total score after introducing each mutation in the variant against the change in total score after introducing each mutation in the WT. Figure S5 displays the change in total score for each interface mutation relative to the WT and all variants for both ACE2 and the NAb. The minimum value (minus 1) is subtracted from each distribution. The effect of each mutation at the interface in the WT is highly correlated for the Delta variant. This correlation is observed for the Gamma and Omicron variants as well, with more significant variation, as expected. There is additionally a small systematic bias toward greater NAb destabilization for mutations with small changes in the total score for the Omicron variant. As discussed in the main text, while this epistatic signature is not observed for the Gamma variant, this trend did not significantly alter the landscape of escape-exacerbating mutations.

### Alternative construction of variant ensembles.

Beginning with the conformational ensemble constructed for the WT, we introduced the variant mutations 452R|478K for the Delta variant, 417T|484K|501Y for the Gamma variant, and 339D|371L|373P|375F|417N|440K|446S|477N|478K|484A|493R|496S|498R|501Y|505H for the Omicron variant and completed 5 rounds of the FastRelaxMover (15 for the Omicron variant) using default parameters (with the exception of disabling design) as described above. The resulting values for the total score and dG separated were comparable to that of the ensembles constructed for the variants beginning with the WT crystal structure (Fig. S16 to S18); however, some structures displayed footprints more similar to the WT than those of the reference ensemble constructed as described in the main text. Thus, this computationally inexpensive alternative may be utilized for rapid evaluation, but the more comprehensive exploration of the conformational space described here is likely desired under many circumstances.

### Data availability.

The data have been deposited through Zenodo (https://doi.org/10.5281/zenodo.5297698), including the supplemental figures and table, and GISAID acknowledgments. Previously published data were used for this work from GISAID (23). Data are additionally made available through FTP site https://ftp.ncbi.nih.gov/pub/wolf/_suppl/SARSstruct21/.
